# Metabolic engineering of *Bacillus subtilis* with an endopolygalacturonase gene isolated from *Pectobacterium*. *carotovorum*; a plant pathogenic bacterial strain

**DOI:** 10.1371/journal.pone.0256562

**Published:** 2021-12-22

**Authors:** Nagina Rafique, Saiqa Bashir, Muhammad Zubair Khan, Imran Hayat, Willium Orts, Dominic W. S. Wong

**Affiliations:** 1 Department of Food Science and Technology, Faculty of Agriculture, University of the Poonch, Rawalakot, Azad Jammu and Kashmir, Pakistan; 2 Bioproducts Research Unit, Western Regional Research Centre, United States Department of Agriculture, Albany, California, United States of America; 3 Department of Plant Breeding and Molecular Genetics, Faculty of Agriculture, University of Poonch Rawalakot, Azad Jammu and Kashmir, Pakistan; University of Poonch Rawalakot, PAKISTAN

## Abstract

Pectinolytic enzymes or pectinases are synthesized naturally by numerous microbes and plants. These enzymes degrade various kinds of pectin which exist as the major component of the cell wall in plants. A pectinase gene encoding endo-polygalacturonase (endo-PGase) enzyme was isolated from *Pectobacterium carotovorum* a plant pathogenic strain of bacteria and successfully cloned into a secretion vector pHT43 having σ^A^-dependent promoter for heterologous expression in *Bacillus subtilis* (WB800N).The desired PCR product was 1209bp which encoded an open reading frame of 402 amino acids. Recombinant proteins showed an estimated molecular weight of 48 kDa confirmed by sodium dodecyl sulphate–polyacrylamide-gel electrophoresis. Transformed *B*. *subtilis* competent cells harbouring the engineered pHT43 vector with the foreign endo-PGase gene were cultured in 2X-yeast extract tryptone medium and subsequently screened for enzyme activity at various temperatures and pH ranges. Optimal activity of recombinant endo-PGase was found at 40°C and pH 5.0. To assay the catalytic effect of metal ions, the recombinant enzyme was incubated with 1 mM concentration of various metal ions. Potassium chloride increased the enzyme activity while EDTA, Zn^++^ and Ca^++^, strongly inhibited the activity. The chromatographic analysis of enzymatic hydrolysates of polygalacturonic acid (PGA) and pectin substrates using HPLC and TLC revealed tri and tetra-galacturonates as the end products of recombinant endo-PGase hydrolysis. Conclusively, endo-PGase gene from the plant pathogenic strain was successfully expressed in *Bacillus subtilis* for the first time using pHT43 expression vector and could be assessed for enzyme production using a very simple medium with IPTG induction. These findings proposed that the *Bacillus* expression system might be safer to escape endotoxins for commercial enzyme production as compared to yeast and fungi. Additionally, the hydrolysis products generated by the recombinant endo-PGase activity offer their useful applications in food and beverage industry for quality products.

## Introduction

In microbial world, the genus *Erwinia* consists of plant pathogenic bacterial species which cause diseases in numerous plants species such as; potato maize, pineapple, and African violet. These bacteria cause diseases in such plants by producing high quantities of several types of cell-wall-degrading-enzymes collectively called exoenzymes such as; pectinases, proteases, cellulases etc. which catalyze the cell-wall-breakdown leading to release of plant nutrients for their growth [1]. Among these bacteria, *Erwinia carotovora*, alsosynonymously known as *Pectobacterium carotovorum*, [2] a gram-negative species belonging to the family *Pectobacteriaceae*, is a plant pathogen to a wide range of agriculturally and economically important plants. It produces pectinolytic enzymes that hydrolyse pectin-polysaccharides within the plant cells. Economically, it is a very important plant pathogen in terms of postharvest losses by causing decay in stored fruits and vegetables [3]. The virulence factors of *Pectobacterium* are grouped under pectinases which include; pectate lyase (*Pel*), pectin lyases (*Pnl*), and pectate hydrolases (*Peh*) also called polyglacturonases (PGs). Polyglacturonase causes widespread maceration of tissue, rotting, and consequently death of the entire plant [3–5]. Although, polyglacturonase mediated enzymatic decay leads to the huge economic loss of crop plants, the specificity of associated infections has received very less attention. Therefore, more studies on pectinases would be of high scientific and economic significance especially for industrial uses.

Pectinases have numerous industrial applications associated with processing of natural products for example; waste water treatment, fruit-juice extraction and clarification, coffee and tea fermentation, vegetable oil-extraction, paper-bleaching in poultry feed additives and various food manufacturing industries [6]. Pectinases are involved in depolymerisation of pectin by its hydrolysis, trans-elimination and de-estrification reactions. Endo-polygalacturonase (EC3.2.1.15), exo-polygalacturonase (EC 3.2.1.67), pectate lyase, pectin lyase and pectin esterase are the well-known pectinases reported in literature [6, 7]. Among these pectinases, endo-polygalacturonase (EndopGase) has been extensively studied for random hydrolysis of α-1, 4 glycosidic bond in the linear chain of pectin [8, 9] Pectin is degraded by cleavage within α-galacturonic acid polymer structure by the action of endo-polygalacturonases (EC 3.2.1.15) [10]. Because of wide applications in food, feed, paper, fruit juice and textile industries, endo-polygalacturonase has gained significant research attention in recent years [6]. Studies on pectinase of microbial bioresources reported 25% of its share for global food and that the industrial enzymes marketing and sales are increasing constantly [11]. Moreover, there is a global market projection of enzymes reaching 6.3 billion USD in the current year 2021 [12]. In food industries, pectinases such as polyglacturonases play a pivotal role for that these are used in extraction of fruit-juices, clarification of wines, cocoa, tea, concentration of coffee; fermentation; extraction of vegetable–oil, pickling and processing of jams and jellies [13, 14]. Additionally, pectinases are used in pulp, paper and fiber industries, treatment of waste-water, poultry-feed additives and biofuels productions [14–16]. Enzymatic catalysis of bioresources is affected by many other factors such as; pH, temperature, nitrogen and carbon source, incubation time, agitation, substrate type and its concentration and utilization of various enzyme formulations during biotechnological processing [15, 17, 18].

Mechanism of catalytic reaction has been well-elaborated through chemical equation earlier for endo-PGAses [19–21]. Many endo-polygalacturonases (endo-PGs) have been recombinantly expressed in *E*. *coli* [22] and also in *Pichia pastoris* [23, 24]. However, bacterial strains other than *E*. *coli* are becoming more applicable for the heterologous protein expression. Gram positive bacillus strains are comparatively remarkable alternative for gene expression than that of *E*. *coli* host. Specially, the *Bacilli* are more beneficial as they have no lipopolysaccharides in the outer layer of cell wall and thus, there is no danger of endotoxins production. Because of high secretion capacity and direct export of protein into medium, *Bacillus* strains have become the most stimulating host system. Among various Bacilli, *B*. *subtilis* is a well-studied prokaryotic strain and considerably used for protein expression [25]. Previously, we cloned and expressed endo-PGase gene from *P*. *carotovorum* into *Pichia pastoris* [26] with encouraging results. Here, for the first time, we report the expression of an endo-PGase gene from *Pectobacterium carotovorum* into *Bacillus subtilis* using pHT43 vectorH. Since *Bacillus subtilis* is promising and well known candidate for the efficient production of industrial enzymes with outstanding characteristics of GRAS (generally recognised as safe strain) supplemented with an easy heterologous expression in culture medium, the researchers around the globe are sharing their efforts for optimizing and enhancing the capabilities of expression system in *Bacillus subtilis*. However, there is no previous report on the expression of EndoPGase which is the widely demanded enzyme, in *Bacillus subtilis* using pHT43 vector system. Hence, we launched this study to express a strong gene of EndoPGase from plant pathogenic strain of pectobacterium into a safe and efficient strain of *Bacillus subtilis* (WB800N) to explore its potential of expression.

The recombinant enzyme was further characterized by performing different molecular and biochemical analyses.

## Materials and methods

### Bacterial strains, plasmids, bacterial culture media and reagents

**Fig 1:**
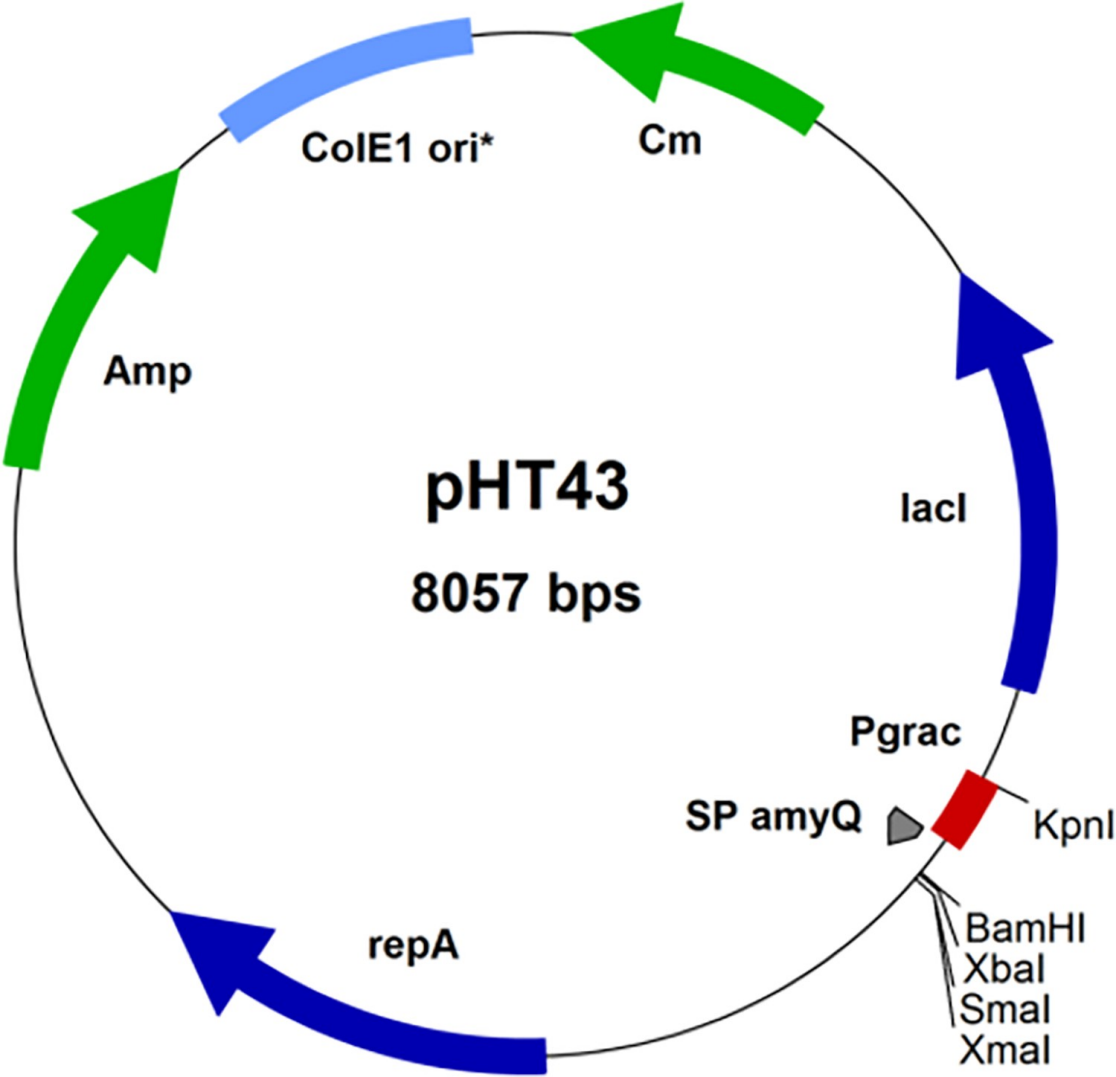
pHT43 an IPTG-inducible expression vector for *Bacillus subtilis*. pHT43 was manipulated for the rapid secretion and purification of recombinant proteins in *Bacillus subtilis*. pHT43 and the eight-fold protease-deficient *B*. *subtilis* strain WB800N, for secretory protein production are available from MoBiTec [27].

The bacterial strains of *E*. *coli* JM109 were purchased from Invitrogen Life Technologies for initial cloning and of endo-PGase (*peh)* gene. For gene expression, *Bacillus subtilis* (WB800N) and its corresponding pHT43 vector (**Fig 1**) for enzyme production and secretion [27] were purchased from MoBiTech (Molecular Biotechnology, GmbH, Germany). Qiagen miniprep kit for recombinant plasmid isolation and purification and SDS-NuPAGE precast gels for molecular characterization of expressed enzyme were obtained from Invitrogen (Carlsbad, CA). Polygalacturonic acid (PGA) sodium salt and citrus peel pectin were purchased from Sigma-Aldrich (MO, USA). Chemicals for culture medium and agar were gathered from Difco Laboratories (Detroit, MI). All other chemicals/reagents used in molecular characterisation, thin layer chromatography (TLC), high performance liquid chromatography (HPLC) and enzyme activity bioassays were of analytical grade.

### Gene synthesis, plasmid construction and transformation of *Bacillus subtilis*

Endo-polygalacturonase *peh* gene was synthesized by Genscript (Piscataway, NJ) using sequence data of the *peh* gene of *Pectobacterium carotovorum* from NCBI and DDBJ. A fragment of 1209bp representing the open reading frame shown in Table 1 was amplified by deducing the desirable gene sequence using the GenBank accession numbers: X52944 genomic DNA translation; CAA37119.1, and L32172 genomic DNA translation; AAA57139.1. The amplified gene fragment was first ligated in the modified pUC57 vector.

**Table 1:** Complementary DNA sequence of ORF of the cloned pehA from *Pectobacterium carotovorum* GenBank accession number X52944 [28].

**ATG**GAATATCAATCAGGCAAGCGAGTTTTATCATTATCACTGGGGCTTATCGGTTTGTTTAGCGCATCGGCATTTGCTTCTGATTCCCGAACGGTGAGTGAACCGAAAGCACCGTCTTCCTGTACGGTGCTTAAAGCTGACAGTAGTACGGCCACGAGTACAATTCAAAAAGCGCTGAATAATTGCGGGCAAGGAAAAGCGGTAAAGCTGAGCGCAGGGAGTTCATCCGTTTTTCTGAGCGGTCCGCTTTCTCTACCTTCTGGCGTGAGCTTATTAATCGACAAAGGGGTAACCCTACGTGCTGTGAATAATGCCAAGTCTTTTGAAAATGCGCCCTCATCCTGTGGCGTGGTGGATACAAACGGTAAAGGCTGCGATGCGTTTATTACCGCCACAAGCACGACGAATAGCGGAATCTATGGGCCGGGCACCATTGATGGACAAGGGGGCGTGAAGCTTCAGGATAAAAAGGTGAGCTGGTGGGATCTGGCTGCCGATGCCAAAGTGAAAAAGTTAAAACAGAATACCCCCCGGCTGATTCAGATTAATAAGAGCAAGAACTTCACGCTGTATAACGTTTCTCTCATTAATTCCCCGAATTTCCACGTCGTGTTCAGCGATGGTGACGGCTTCACCGCGTGGAAAACCACGATCAAAACGCCATCTACCGCCAGAAACACCGACGGTATCGATCCTATGTCGTCAAAGAACATCACCATTGCCCACAGTAATATTTCGACGGGTGACGATAATGTGGCGATCAAAGCCTATAAGGGGCGTTCTGAGACACGCAATATTTCCATTTTGCATAATGAATTTGGAACGGGACATGGCATGTCGATCGGTAGCGAAACGATGGGAGTTTATAACGTGACGGTCGATGATCTGATTATGACTGGCACCACGAATGGCTTACGTATTAAAAGCGACAAATCAGCAGCTGGCGTTGTCAATGGTGTTCGGTATAGCAACGTAGTCATGAAGAACGTGGCGAAACCGATCGTGATTGACACGGTATATGAGAAAAAAGAGGGAAGTAATGTTCCTGACTGGAGCGACATTACGTTTAAGGATATTACGTCTCAAACCAAAGGCGTGGTGGTGCTGAACGGCGAGAATGCGAAAAAGCCGATAGAAGTGACGATGAAGAACGTCAAACTGACGAGCGACAGCACATGGCAAATCAAGAACGTCACCGTTAAGAAG**TAG** *

**Table 1** *ATG is the start codon shown bold red whereas; codon with asterisk represents the stop codon.

Then the sequenced gene was subcloned in fusion to the amyQ signal peptide downstream of the p_*grac*_ promoter in *Bacillus* pHT43 vector (MoBiTec GmbH, Germany) using codon optimisation approach following the manual supplied by MoBiTec GmbH (Göttingen) [27].

Briefly, the endo-PGase gene was PCR amplified and a secretion vector pHT43 specific to *Bacillus subtilis* strain WB800N was constructed based on σ^A^-dependent promoter P*grac*. The constructed vector consisted of *gro*E promoter, SD_*gsiB*_ gene sequence and a SP_*amyQ*_ (*Bacillus amyloliquefaciens* signal peptide) with multiple cloning site (*Bam*HI, *Xba*I, *Aat*II and *Sma*I) and *lac*O operator. The expression vector was transformed into *E*.*coli* JM109 before transforming into *Bacillus subtilis* following the instructions from manual provided by MoBiTec GmbH, Germany. The transformed colonies were screened by double digestion of the isolated plasmids from overnight grown cultures and the positive transformants were confirmed by agarose gel electrophoresis and DNA sequencing simultaneously.

### Genetic techniques: Preparation of competent cells

*Bacillus subtilis* competent cells were prepared by inoculating 50 ml HS medium with overnight grown culture of appropriate recipient cell line of *Bacillus subtilis* and incubated at 37°C shaker incubator. The growth was recorded after every 40 min and at stationary phase 10 ml of sample was taken with the interval of 15 min, sterilized glycerol stock (87%) was added and placed on ice for 15 min. The entire sample was fractionated into aliquots of 1 ml and was frozen using liquid nitrogen following the instruction manual supplied by MoBiTec GmbH, Germany (available online). The prepared stocks of competent cells (*Bacillus subtilis* WB800N) were stored at -80°C freezer. Desirable stocks quantities were shifted to -30°C freezer and gradually thawed in ice for transformation by recombinants as and when scheduled following the protocol supplied by MoBiTec GmbH (Göttingen) [27].

### Recombinant DNA techniques: Expression of recombinant endo-polygalacturonase

The enzymatic protein was expressed by inoculating fresh liquid culture at OD 0.8 with 1mM IPTG following the second induction of IPTG which was done after 8 hrs of the first induction. The culture was grown at 37°C in a shaking incubator at 225 rpm. Endo-polygalacturonase production from recombinant *Bacillus subtilis* was first tested in 250 ml flask with 50 ml of 2X-YT medium. Cell free culture supernatants were harvested by centrifugation after 24h of the first induction and up to 120 h, the samples were continuously collected after every 24 h and assayed for endo-PGase activity following dinitrosalicylic (**DNS) method** [29].

### Biochemical characterization of recombinant endo-PGase

In order to determine the optimal pH and temperature requirements of the recombinant endo-PGase, the enzyme activity was measured at pH ranging from 4.0–10.0 and temperature ranging from 20°C—70°C. For estimation of pH stability, the recombinant enzyme samples were incubated with buffers of different pH at 25°C for 4 hrs and then the residual activity was measured simultaneously. Thermal stability was evaluated by incubating the enzyme samples at temperatures ranging from 20°C to 70°C in 0.2M acetate buffer of pH 5.0 for 45 min and residual activity was measured by DNS method following Wong et al [29]. Effect of five different metal cations was determined by incubating the enzyme samples with 1 mM concentration of each of the metal ion i.e., Ca^++^, Mg^++^, EDTA, Zn^++^ and K^++^. At the same time, one control sample without having any metal cations was also included as blank. The enzyme activity was again measured by DNS method as previously described [26, 29].

### Molecular analysis of recombinant endo-PGase gene expression

Cell free culture supernatant was analysed for recombinant protein expression with SDS-PAGE electrophoresis system. SDS-PAGE gel electrophoresis of cell free supernatant was carried out using Novex mini gel system (Invitrogen) with Nu-PAGE 10% Bis-Tris gels and MES-SDS running buffer. A known molecular weight (kDa) protein standard marker was loaded in the first well to quantify the unknown weight of the recombinant enzyme samples loaded in the adjacent wells. Finally, the gel was stained with Coomassie Brilliant Blue, destained and then photographed.

### Enzyme activity analysis

Culture supernatant was tested for enzyme activity using solid as well as liquid assay methods. For plate assay, the aliquots of enzyme were inoculated into wells on plates containing 0.5% pectin (from citrus peel) and 0.5% agarose. Plates were incubated overnight at 37°C and stained with 0.02% ruthenium red. Liquid assay of culture supernatant was performed using DNS method to measure the galacturonic acid produced [29] The reaction mix contained 75 μl of polygalacturonic acid (PGA) sodium salt (1%), 75 μl of 0.2M sodium acetate buffer of pH 5.0 and different concentrations of enzyme. The reaction mix (pH 5.0) was incubated at 40°C for 1 h and unit of enzyme activity was calculated as μg of galacturonic acid/min at 37°C temperature.

### TLC and HPLC analysis of hydrolysis products

Analysis of hydrolysis reaction products of PGA- sodium salt was performed using thin layer chromatography. An aliquot of 10 μl reaction was spotted onto TLC plates (20 x 10 cm silica plate) with mobile phase containing 2:1:1 ratio of ethyl acetate/acetic acid/water. The hydrolysis reaction products and standard oligo-galacturonates of different pectic substrates were analysed by HPLC using Zorbax-SAX column (200 ×10.0 mm, Agilent) in 0.3M sodium acetate (pH 5.0) at a flow rate of 0.9 ml per min at 40°C [30]. The injection volume of 5 μl was applied to the injector and monitored by detector. Organic acids peaks were screened using refractive index detector.

### Bioinformatics and molecular graphics of the cloned endo-PGase gene

For recombinant vector construction and sequence analysis, Geneious was used [31]. Multiple sequence alignment was performed using by ClustalW and graphicalyl presented by BioEdit Sequence Alignment Editor 7.2 [32]. Evolutionary analyses were conducted in MEGA (Molecular Evolutionary Genetics Analysis) 7 [33]. The evolutionary history was inferred using the Neighbor-Joining method [34]. The evolutionary distances were computed using the Poisson correction method [35] and are in the units of the number of amino acid substitutions per site. The analysis involved 9 amino acid sequences. All positions containing gaps and missing data were eliminated. There were a total of 342 positions in the final dataset. 3D structure of EndoPGase enzyme was visualized with the Phyre2 web portal for protein modeling, prediction and analysis [36].

### Statistical analysis

For plotting graphical figures and calculations of standard bars **Kaleidah Graph software** was used [37].

## Results and discussion

### Cloning, isolation and characterisation of *PehA* gene

**Fig 2:**
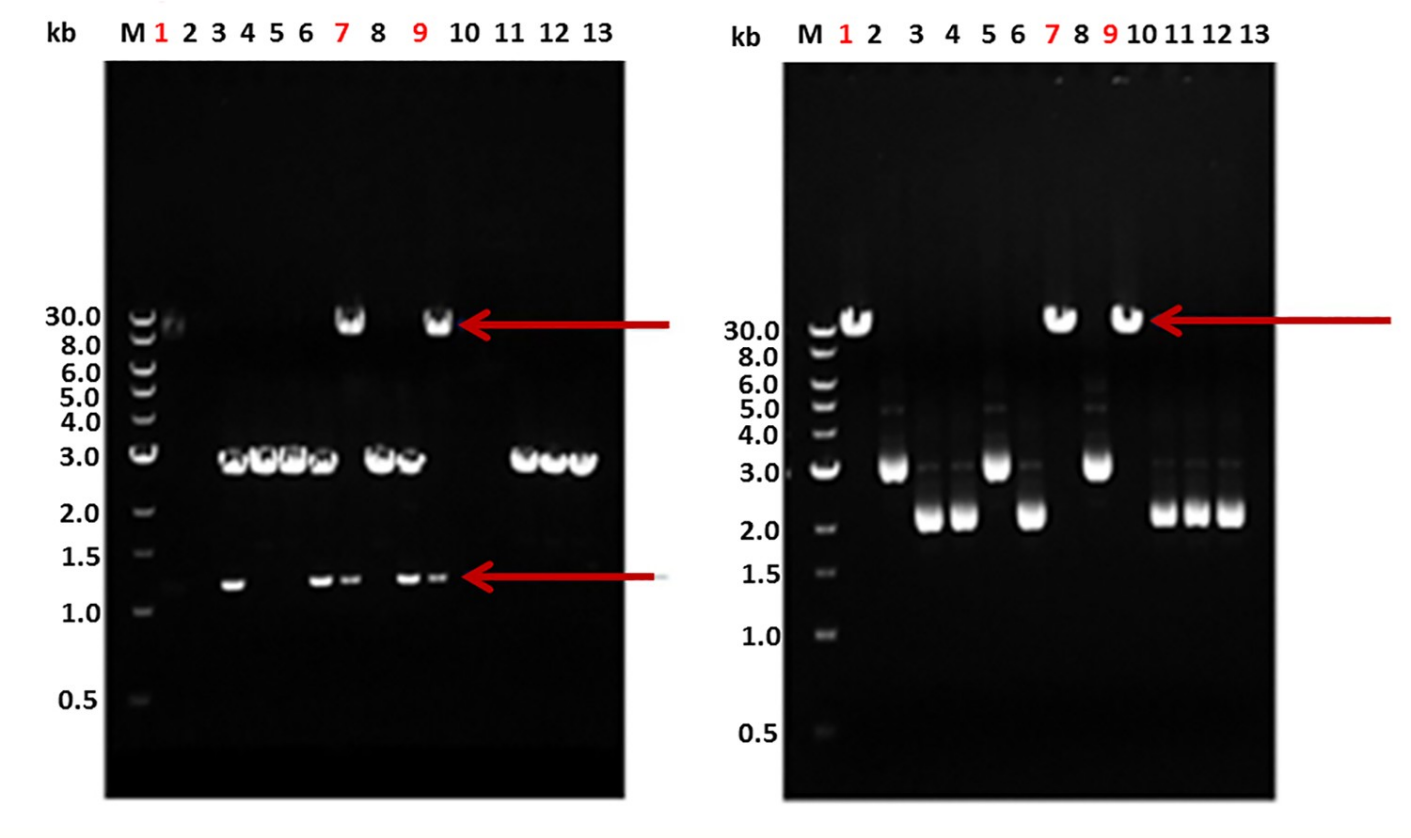
Horizontal gel electrophorogresis of the cloned *PehA* gene from *P*. *carotovorum*. Double digestion with BamHI and XbaI for the clone confirmation of transformed colonies (left). Restriction digestion with Cla Lane 1, 7 and 9 represents the clone confirmation of our gene of interest (right).

The desired PCR product was **1209bp,** first recovered by agarose gel electrophoresis (**Fig 2**). The isolated gene was further confirmed by sequence analysis.

**Fig 3:**
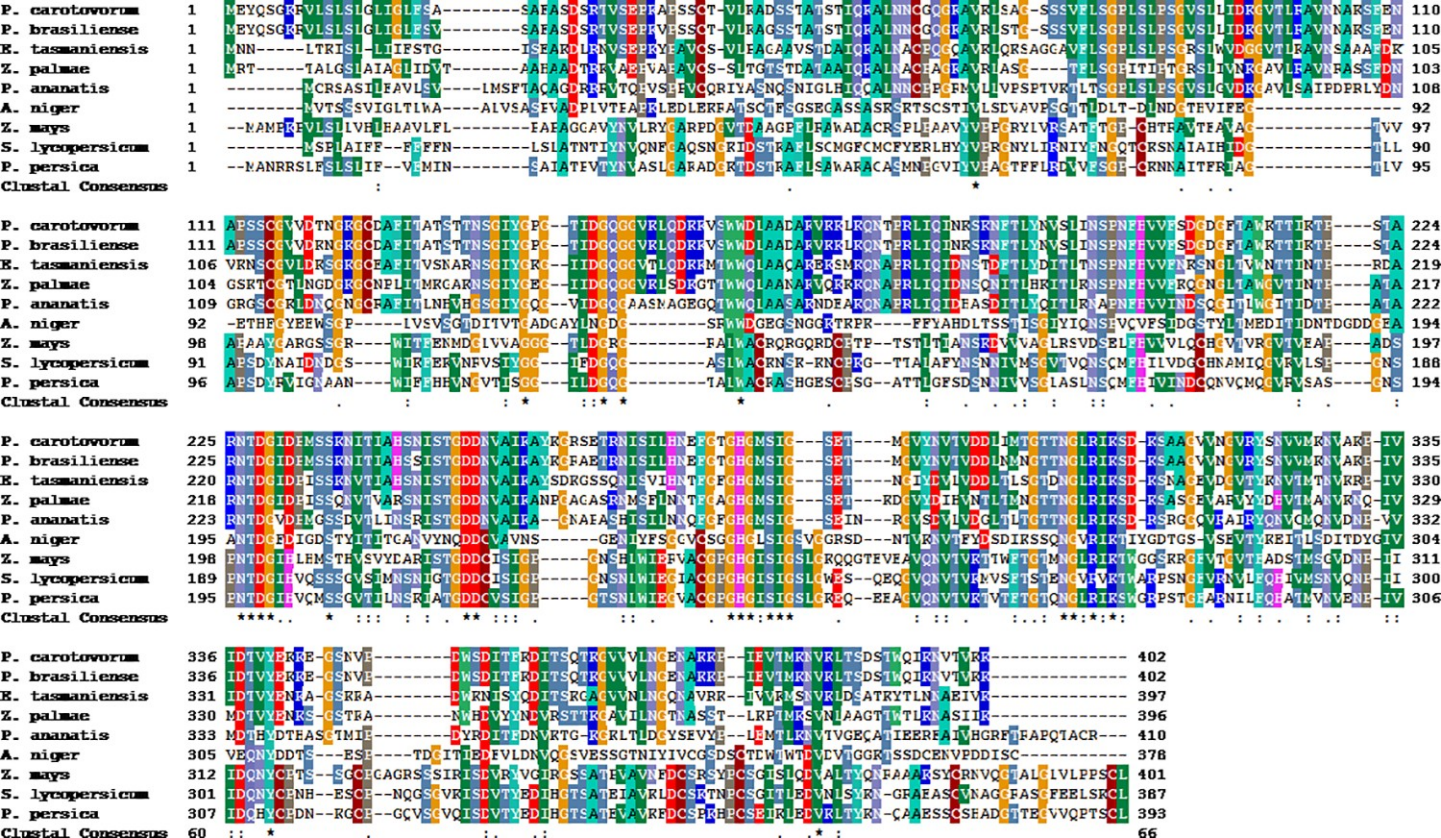
Multiple sequence alignment of *PehA* gene with the selected taxa. (from top to bottom); *Pectobacterium carotovorum*, *Pectobacterium brasiliense* (WP_039510994.1), *Erwinia tasmaniensis* (WP_042958805.1), *Zymobacter palmae* (WP_027705705.1), *Pantoea ananatis* (PA13 AER33559.1), *Aspergillus niger* (CAA74744.1), *Zea mays* (ACF85710.1), *Solanum lycopersicum* (AAB09576.1) and *Prunus persica* (AEI70578.1).

The open reading frame of cloned gene confirmed the encoded polypeptide of 402 amino acids. The signal peptide, as confirmed from Phyre2 showed signal peptide of 26 amino acid residues with the N-terminus. BLAST, PDB and Uniprot search for the amino acid sequence revealed close evolutionary relation of it with the isolated gene. Multiple sequence alignment of related EndoPGase across selected microbial and plant genera with the isolated gene is shown in (**Fig 3**) which showed sequence identity of 97.51% with *Pectobacterium brasiliense* (WP_039510994.1), 62.50% with *Erwinia tasmaniensis* (WP_042958805.1), 57.65% with *Zymobacter palmae* (WP_027705705.1), 50.00% with *Pantoea ananatis* (PA13 AER33559.1), 32.54% with *Solanum lycopersicum* (AAB09576.1), 32.37% with *Zea mays*, (ACF85710.1), 31.50% with *Prunus persica* (AEI70578.1) and 28.23% with *Aspergillus niger* (CAA74744.1) respectively.

The present results of cloning and characterisation of *PehA* from *P*. *carotovorum* are identical to those previously presented by Hinton et al [28] who cloned and isolated a *pehA* gene from the same strain which encoded a polypeptide comprising 402 amino acid residues In our previous report, the nucleotide sequence of the open reading frame (ORF) of *PehA* gene isolated from *P*. *carotovorum* showed the sequence identity of 61.5% with *Erwinia tasmaniensis* (WP_042958805.1), 57.8% with *Zymobacter palmae* (WP_027705705.1), and 50.00% with *Pantoea ananatis* (PA13 AER33559.1) respectively [26] which are identical to our present results. However, differences in identities among genomic sequences have been observed that are the primary sources of variation even among the members of the same species. The degeneracy of the codons is the reason why most of the genes encode similar amino acid sequences for a particular protein.

Phylogenetic analysis of the deduced polypeptides encoded by the isolated *PehA* genes when compared to other known EndoPGase genes across bacterial, fungal and plant genera revealed that the *PehA* of *P*. *carotovorum* individually positioned closer to the corresponding EndoPGase of *Pectobacterium brasiliense*. Both *P*. *carotovorum* and *Pectobacterium brasiliense* closely positioned with *Erwinia tasmaniensis* since, all these bacteria belong to the same genus. Similarly, in case of higher plants, *Solanum lycopersicum* and *Prunus persica* positioned close to each other despite of belonging to different families and mutually linked closer to *Zea mays*, which is a cereal crop. Surprisingly, *Aspergillus niger* was neither closely related to bacterial species nor the higher plants. It indicates that PGs might have diverged long before the divergence of species within Aspergillus. Further, it has been reported that Aspergillus species have evolved as well as diverged over 200-millions of years and whole-genome-duplication and subsequent gene loss preceded the speciation of Aspergillus during evolution of eukaryotes [38].

**Fig 4:**
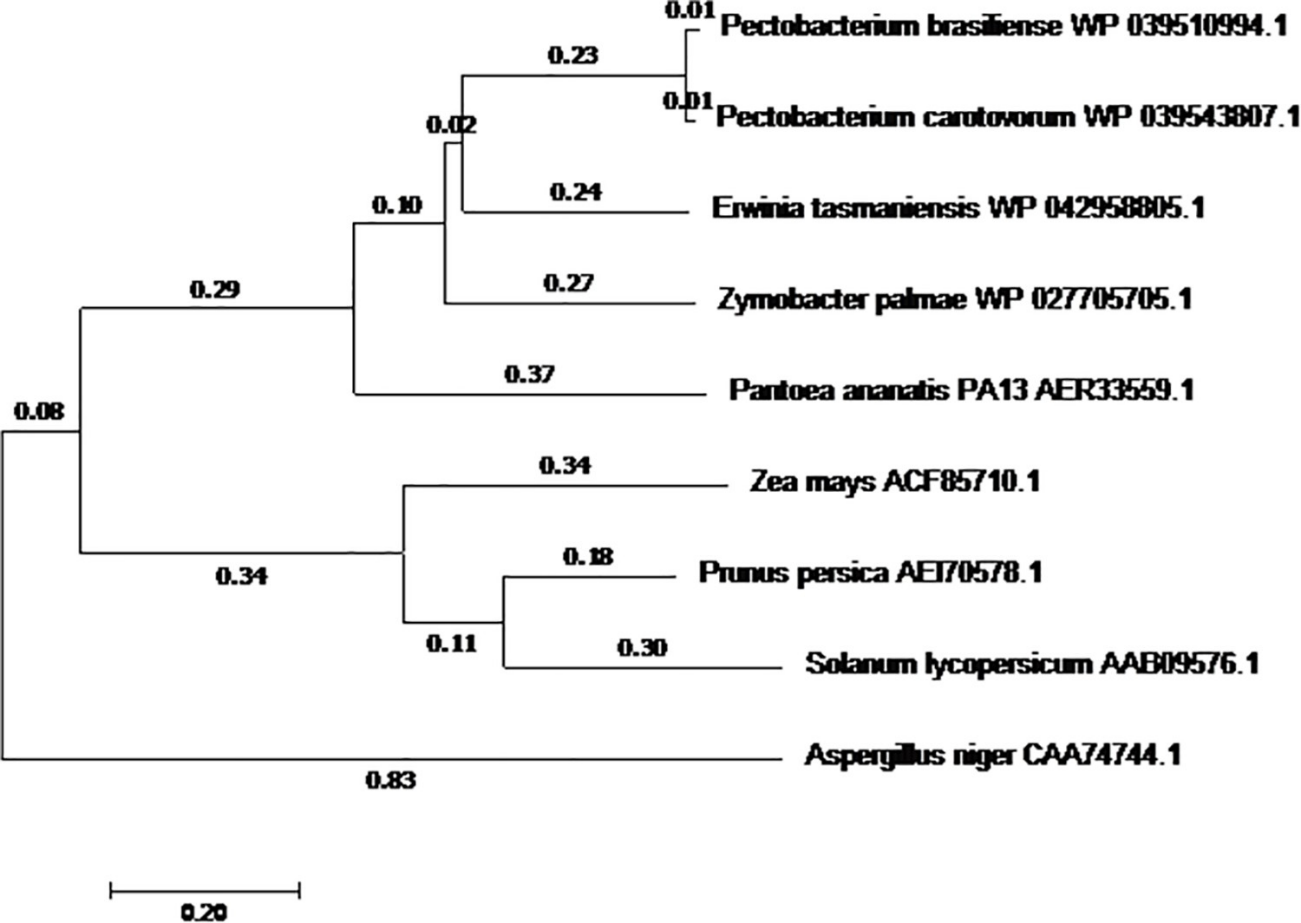
Evolutionary relationships of taxa across microbial and higher plant species for EnoPGase. The values show the evolutionary genetic distances among the species.

Similar results were previously presented by Yadav et al [39] who aligned some 48 full-length protein sequences of pectin lyases from various organisms and generated phylogenetic trees. They found pectin lyases from bacterial and fungal species bifurcating into two distinct clusters which suggest that the bacterial *PehA* are more desirable for transformation of higher plants because of closer evolutionary relation. The optimal tree with the sum of branch length = 3.71811606 is shown in (**Fig 4**). The tree is drawn to scale, with branch lengths in the same units as those of the evolutionary distances used to infer the phylogenetic tree.

**Fig 5:**
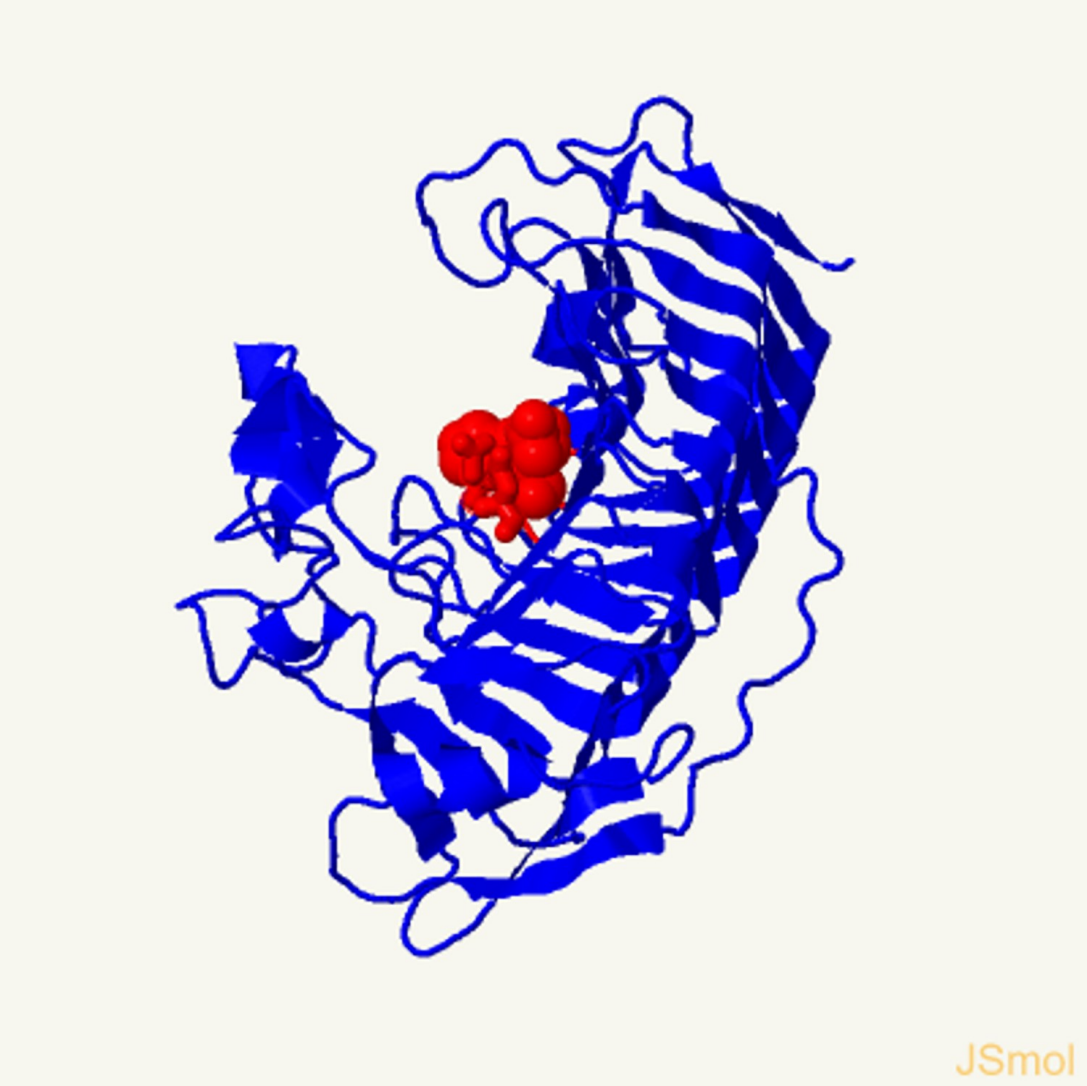
3D structure of the EndoPGase encoded by the corresponding *PehA* gene expressed in *B*. *subtilis*. A single-stranded right-handed beta-helix view analysed by Phyre2 and the residues with active site of the enzyme are highlighted red.

3D structure of Isolated EndoPGase was analysed by Phyre2 and is shown in Fig 5. For production of recombinant proteins, *Bacillus subtilis* is an attractive host as it is a well-known non-pathogenic organism. *Bacillus subtilis* is considered as GRAS and also large information related to genetic manipulation, protein expression mechanism and fermentation on large scale has been developed for this organism and it will be safer to use in food industries. Hemilä et al [40] expressed the above mentioned *PehA* gene in *Bacillus subtilis* by using a secretion vector with signal sequence and promoter of the gene (*amyE*) encoding α-amylase from *Bacillus amyloliquefaciens*. They found that all of the Bacillus strains harbouring the recombinant constructs secreted endo-PGase directly into the growth medium and there was about fourfold increase in the production efficiency of *B*. *subtilis* strains carrying various plasmids constructs In our study, we used the pHT43 secretion vector to assess the expression efficiency in *Bacillus subtilis* and an increased expression was observed.

**Fig 6:**
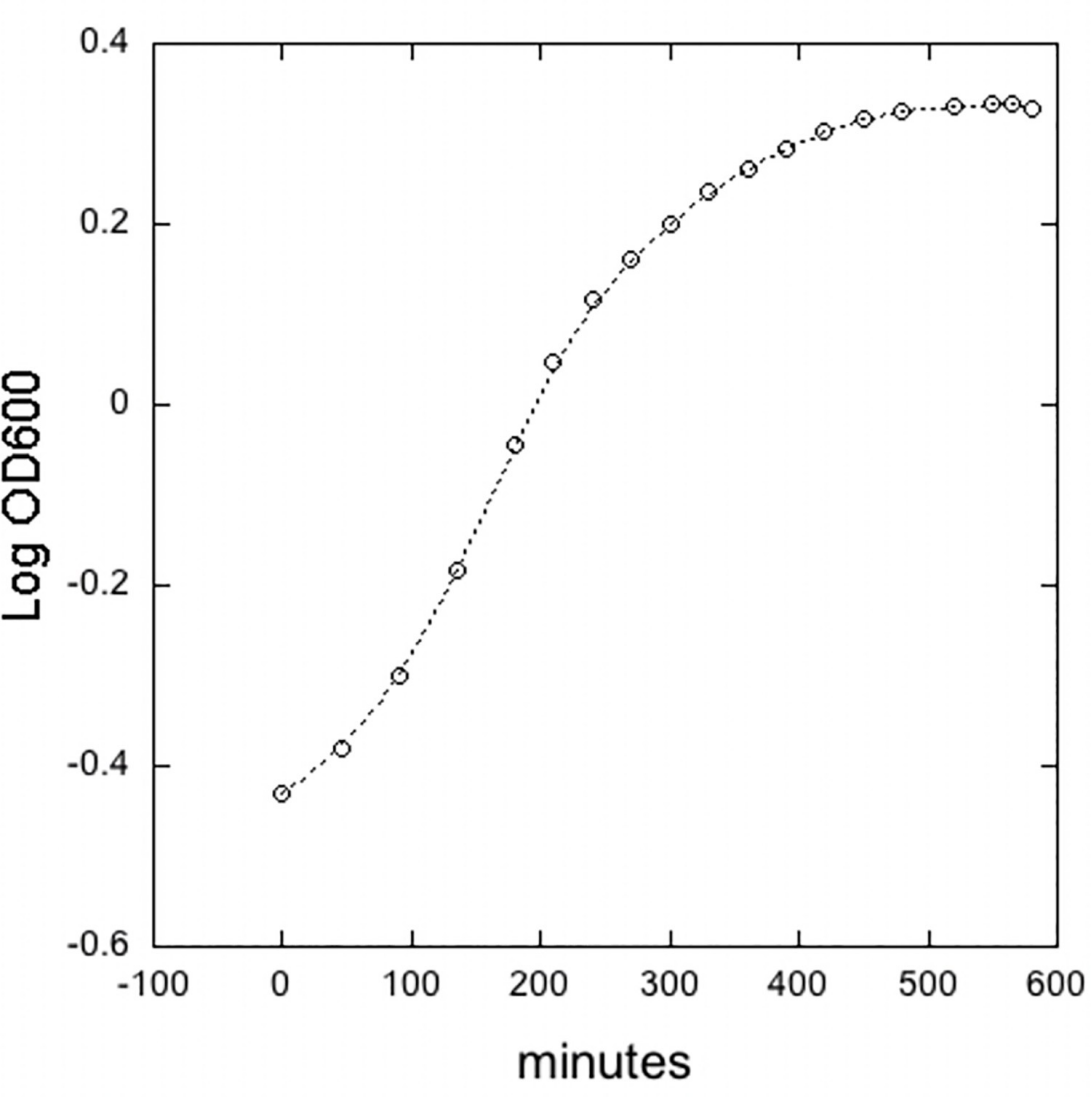
Growth curve of *Bacillus subtilis* in 2X-YT medium. Optical density was measured at 600 nm using microplate. Three sets of competent cells were prepared at stationary phase after every 15 minutes of interval.

**Fig 6** represents the growth curve of protease deficient strain of *Bacillus subtilis* (WB 800N) by measuring the optical density of bacterial culture. The growth curve was obtained after measuring the absorbance values of the bacterial culture at different time intervals. For preparation of competent cell of *Bacillus subtilis* (WB 800N), stationary phase of growth is required to capture. It has been observed that under optimal conditions, bacillus doubles the growth in 30 minutes which represents the logarithmic growth phase followed by a stationary phase of growth [27]. It was observed that the WB 800N becomes competent at both early and the stationary growth phases.

**Fig 7:**
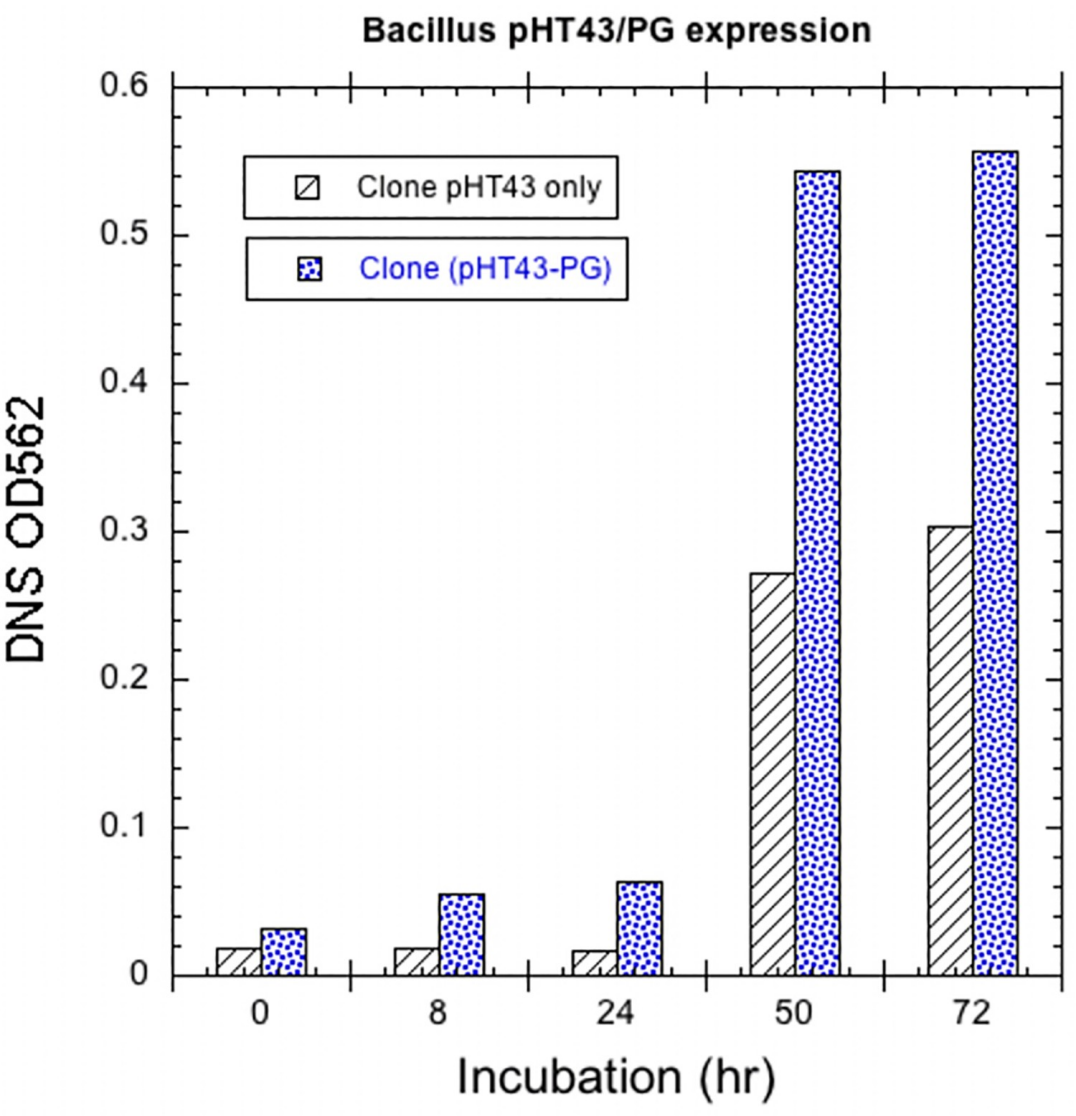
Expression recombinant endo-PGase in 2X-YT culture of *Bacillus subtilis*. Medium was initially induced with 10 μl of 0.5 mM IPTG when culture OD reached at 0.8 after 2hrs of inoculation and again induction was done after 8 hrs of the first induction. Activity of both control (vector clone) and clone (vector with PG gene) was checked after at different time intervals using DNSA method.

The comparison of initial expression of the recombinant gene clones of *Bacillus Subtilis* (WB800N) with those harboring the empty pHT43 vector by IPTG induction is shown in **Fig 7**. The clone containing a pHT43 vector with Endopolygalacturonase gene showed significant enzyme expression after 72 hours while vector without gene only showed a minimal production of reducing sugars because any bacteria can produce reducing sugars by metabolizing medium ingredients when assayed with DNSA method.

**Fig 8:**
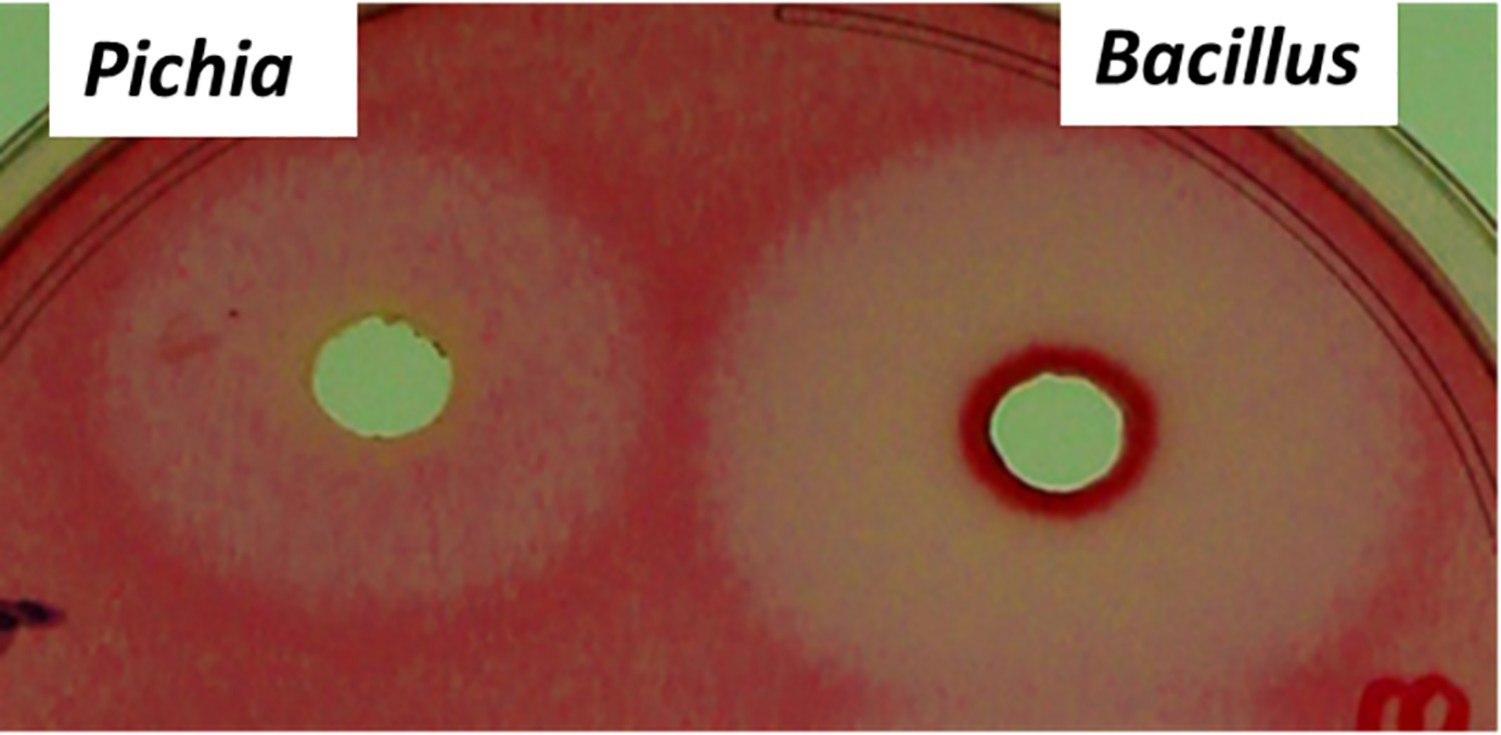
Enzyme activity by plate assay. For preparing plate a mixture of 0.8% agarose and 0.5% of citrus pectin in sodium acetate buffer of pH 5.0 and dissolved in microwave. Positive control (*Pichia* expression) and *Bacillus* enzyme were filled in the wells, incubated overnight at 37°C and stained with 0.02% ruthenium red.

**Fig 9:**
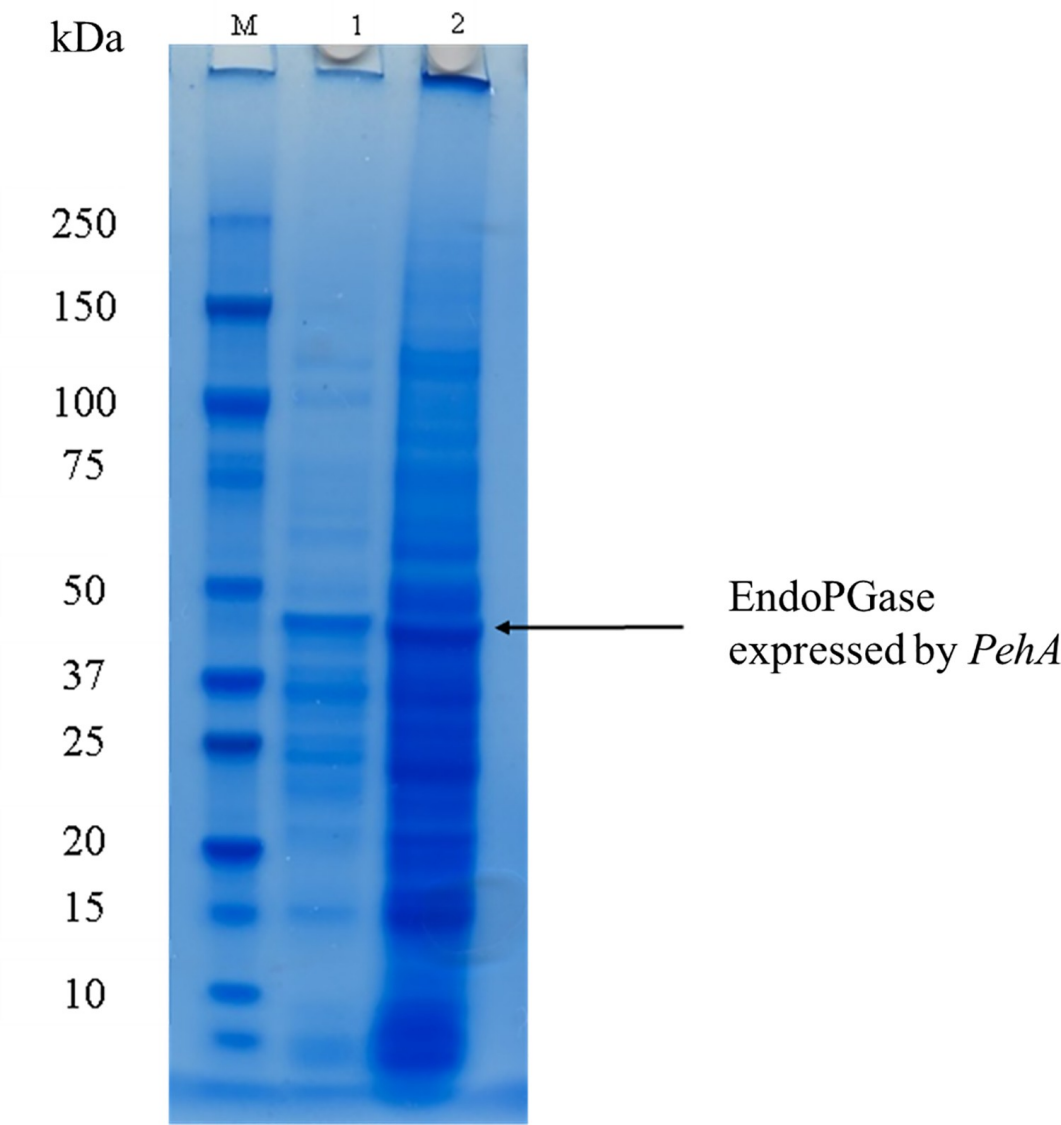
SDS-PAGE analysis of recombinant endo-PGase. Lane 1. Protein marker. Lane 2. Enzyme sample without concentrating. Lane 3. endo-PGase concentrated using Amicon ultra (10K NMWL).

The appearance of pink halos with transparent background on polygalacturonic acid plate and clear holes on citrus pectin plates confirmed the activity of recombinant enzyme as shown in **Fig 8**. After incubation of substrate containing agarose plate with the recombinant enzyme plates were stained with ruthenium red dye hence, the hydrolysed substrate captured the colour of dye and resulted in the appearance of pink halos.The non-concentrated and concentrated recombinant proteins were further subjected to SDS-PAGE analysis for molecular identification which revealed a molecular mass of 48kDa (**Fig 9**), corresponding to the molecular mass of endo-PG gene encoding wt-PehA (47 kDa) shown by Massa et al [22]. Previously, expression of a polygalacturonase encoding *PehA* gene from *Pectobacterium carotovorum* in *E*. *coli* was analysed by Ibrahim et al [41] who reported the molecular mass of 41.5 kDa which is lower than to that found by us. Previously, a p*ehA* gene encoding endopolygalacturonase expression in *Pichia pastoris* was presented by Liu *et al* [24] which exhibited a molecular weight of 40 kDa.

Another endo-polyglacturonase gene encoding endo-pga from *Aspergillus niger* was cloned and expressed in *Saccharomyces cerevisiae* EBY100 [42] which showed a molecular mass about 38.8 kDa.Several other reports on cloning and expression of endo-PG into *Pichia pastoris*, other yeasts, and fungi have been published previously. Sawada et al [43] described the cloning of a gene *pehK* encoding exo-Pgase and its expression in *Bacillus subtilis*. Since the ultimate goal in metabolic engineering is to use the biological systems in order to synthesize a desired protein encoded by a related gene of interest hence, the whole process itself is known as the engineered-protein-expression. Once the target protein or enzyme synthesized by gene engineering methods shows all the structural and functional characteristics of naturally existing protein, the achievement of the cloning target is confirmed. On the other hand, when a metabolic engineer tries to synthesize large quantities of a specifically targeted protein or enzyme synthesis and isolation of which from natural sources might be a hectic process, the heterologous expression itself becomes the end of the whole experiments [44]. Here for the first time we reported cloning and expression of a gene encoding endo-Pgase in *Bacillus subtilis* from *Pectobacterium carotovorum*.

### Biochemical characterization of recombinant enzyme activity

**Fig 10:**
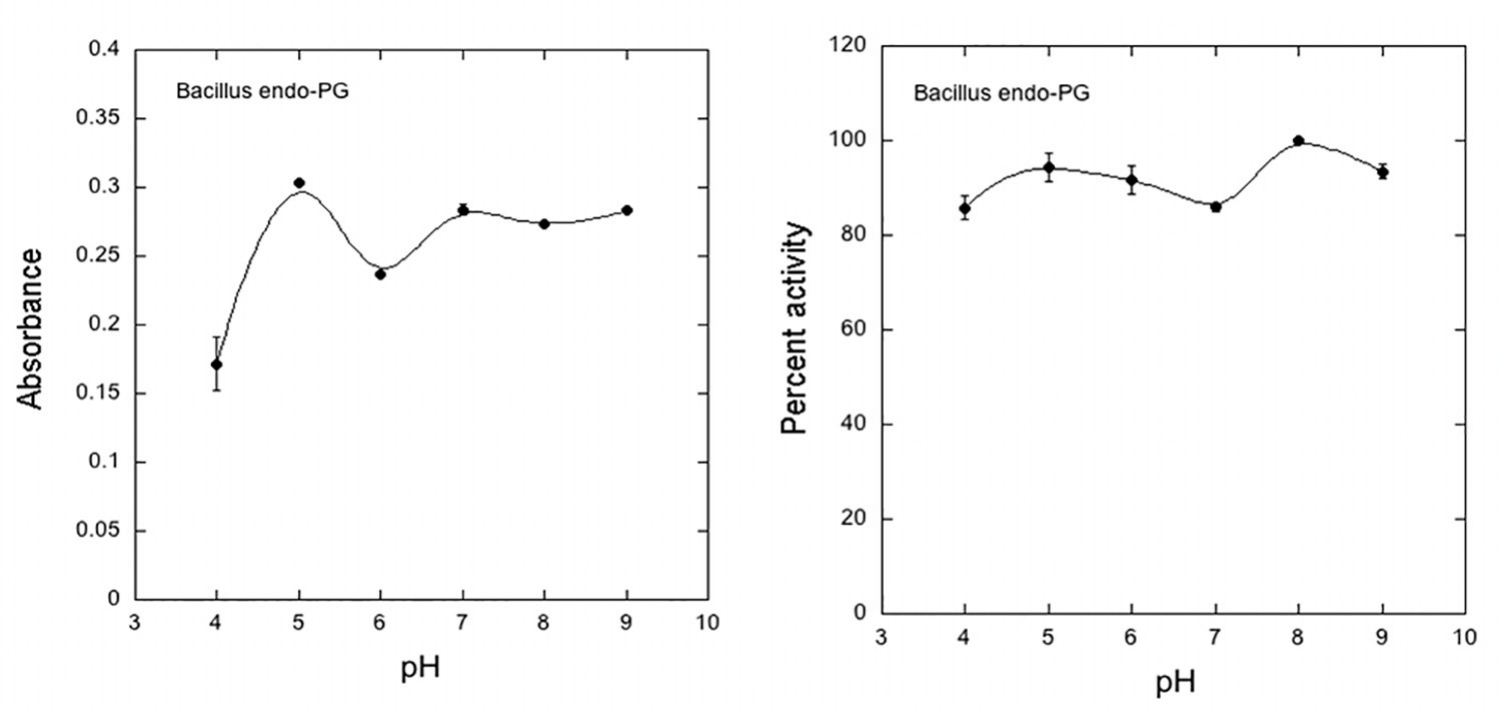
Effect of pH for maximum enzyme activity. **(a)** Enzyme samples were incubated at 40°C for 1 hr in 0.5% PGA as substrate in different pH buffers. **(b).**Effect of pH on enzyme stability. Enzyme stability was measured by pre-incubation of sample aliquots in various pH buffers at 25°C for 4 h, then pH was readjusted t to 5.0 and assayed for present activity.

The recombinant enzyme was characterized for optimal pH (**Fig 10(A)** and the results showed maximal enzyme activity at pH 5.0. and displayed stability over an extensive pH range from 5.0 to 9.0 **(Fig 10(B)**. Previous reports have shown that pectinolytic enzymes show higher enzymatic activity at pH range of 4.0 to 7.0 [15].

**Fig 11:**
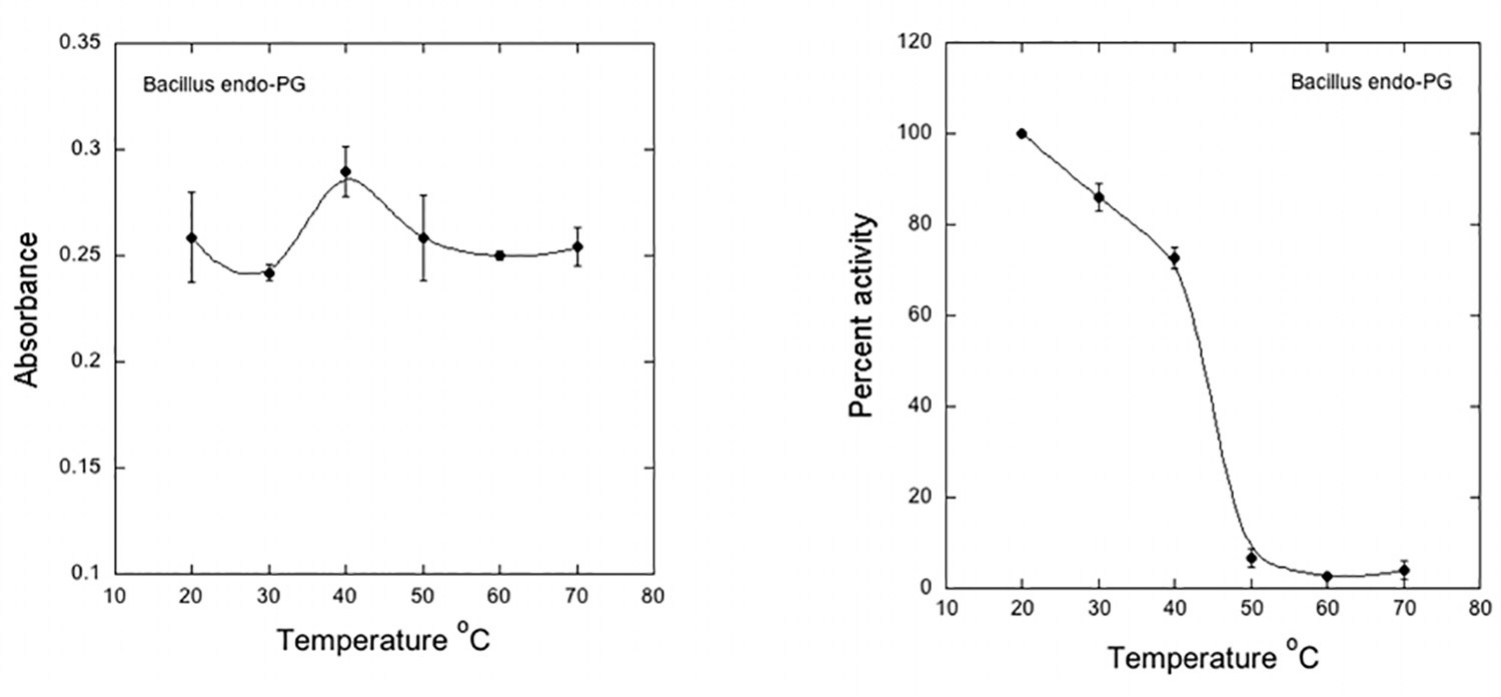
Effect of temperature on enzyme activity. **(a)** The substrate and enzyme mixture was incubated at different temperatures for 1 h in 1 mM Sodium acetate buffer (pH 5.0). **(b)**. Effect of temperature on optimum enzyme stability. The enzyme was incubated at various temperatures for 25 min before starting enzyme assay and then the residual activity was measured by conducting standard enzyme assay.

Optimum enzyme activity was observed at a temperature of 40°C. **Fig 11(A).** However, the recombinant enzyme showed an overall decline in stability within the temperature range from 20°C to 70°C **Fig 11(B)**. The recombinant endo-PG retained 80% of its activity at 30°C while more than 70% activity was restored at 40°C. Zhou et al [42] characterized an endo-PgaA from *Aspergillus niger* and expressed in *Saccharomyces cerevisiae*. The recombinant PGase was found active at pH 5.0 and showed a pH stability range within pH 3.0–6.0. The enzyme was active at 50°C and retained its activity for 60 minute incubation but no activity was observed after 2 h of incubation at 70°C confirming the temperature specificity for catalytic activity normally exhibited by other endoPGs of bacterial or fungal origin. These findings suggest the higher catalytic affinity and efficiency of recombinant EndoPGase to pectin. Our results confirm the previous findings that the induction, production and specific function of an enzyme which is produced inside a cell and is secreted to the outside environment, can take place only under some optimal pH [3]. It has been reported through numerous studies that pectinases show enhanced catalytic activity at a pH ranging from 4.0 to 7.0 [15].

### Effect of metal ions

**Fig 12:**
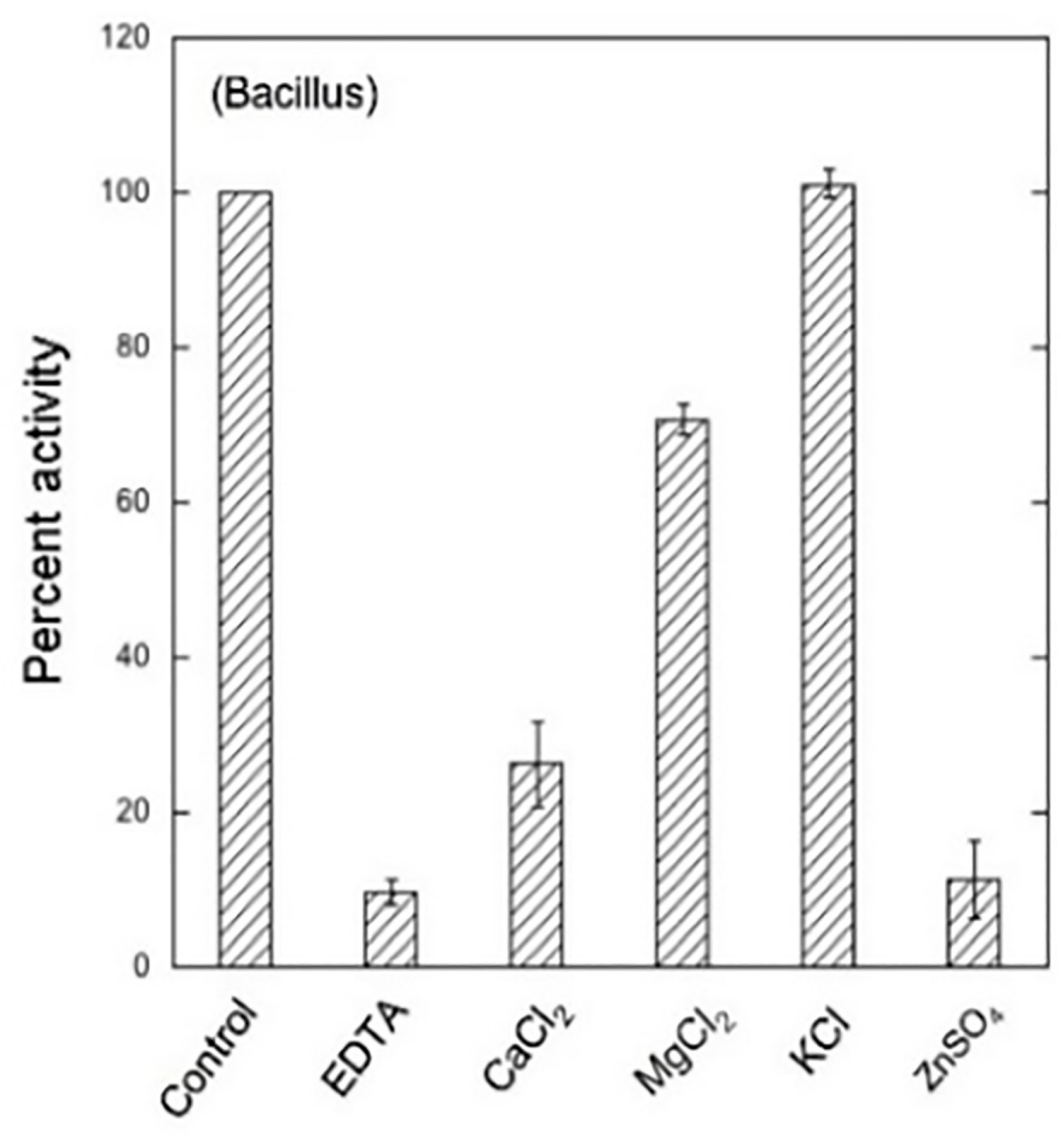
Effect of metal ions on enzyme activity. I mM concentration of metal ions was used in pre-incubation of enzyme-metal mixture at 4°C for 1 h and residual enzyme activity was measured under standard assay conditions at 40°C.

The presence of potassium chloride increased the enzyme activity while EDTA, Zn^++^, Ca^++^, strongly inhibited the activity (**Fig 12**). Previously, Li et al [45] reported that 1 mM EDTA had no effect on the activity while the same concentration of Ca^++^ was observed to decrease its relative activity. Since K++ and Mg++ can inhibit the enzyme activity by causing precipitation but recombinant enzymes are usually more stable and the interaction time for both of these metal ions might be less than the required for inhibition so these metal ions inhibit or did not show any negative effect on enzyme activity.

An-other report showed inhibition in enzyme activity with the addition of Mg, Zn and Ca [46]. Similarly, the activity of endo-polygalacturonase from *Bispora* sp. MEY-1 [47] expressed in *Pichia pastoris* was strongly inhibited by the addition of EDTA, Zn^++^, Ca^++^, SDS and remained un affected by K^++^ and Mg^++^. Enzymes require metal ions for catalytic activities and to maintain the stability of their structure under severe conditions of surrounding environment. However, depending upon the fine structure and the source, the endo-PaGs show a wide range of results in interaction with the metal ions.

### HPLC and TLC analysis

**Fig 13:**
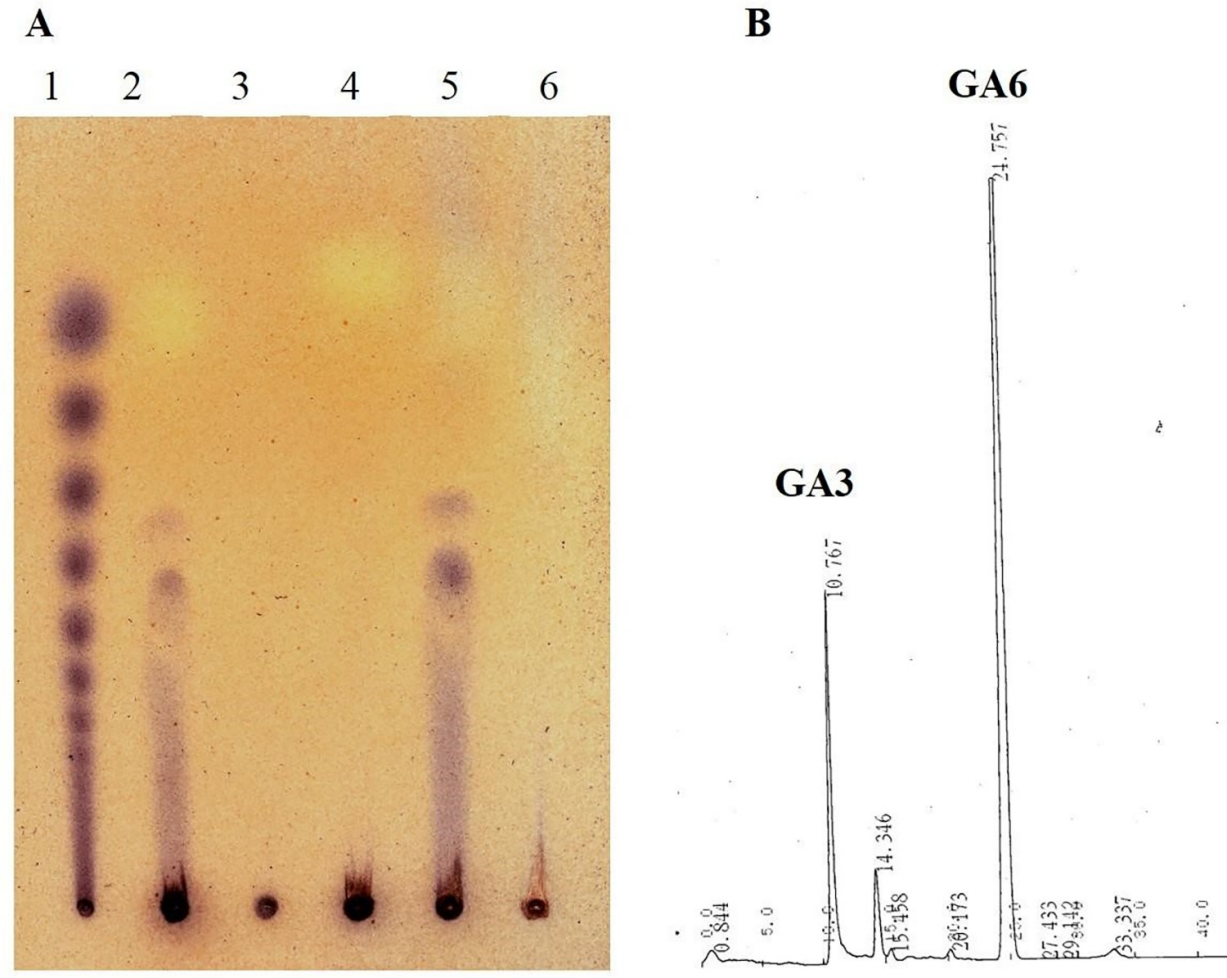
TLC analysis of oigogalacturonates produced by the enzymatic hydrolysis of PGA substrates. **(a)** Lane.1.TLC standard, Lane. 2. Enzyme + 0.5% PGA, Lane. 3. Enzyme, Lane. 4. PGA 0.5% + Na-acetate buffer, Lane 5, EndoPGAse + PGA Lane 6, Control. Lane 2 and Lane 5 show the tri and tertra-galacturonates released as a result of enzymatic hydrolysis. **(b).** HPLC chromatogram showing peaks of tri and tertra-galacturonates as the end products of hydrolyses by the recombinant EndoPGase.

The hydrolyzate of PGA and pectin was analyzed by HPLC and TLC. The reults of TLC revealed that tri and tetra-galacturonates were the end products of substrate hydrolysis (**Fig 13(A).** This evidenced the endo-acting action of the recombinant enzyme. While the complex/higher oligosaccharides form a smear on TLC plate and were not further degraded into smaller oligosaccharides. In our previous study, recombinant endo-PGase enzyme when heterologously expressed in *Pichia pastoris*, hydrolysed PGA substrate into tri and hexagalacturonates [26] however, in the present analysis, HPLC chromatograms (**Fig 13(B)** obtained from the products of hydrolysis by recombinant EndoPGase showed unique peaks which confirmed the presence of tri and tertra-galacturonates. In comparison with our current results the recombinant endopolygalacturonase from *Pichia pastoris* and *Saccharomyces cerevisiae* yielded mono, di, and trigalacturonic acids, as the end products of hydrolysis [24, 42]. These findings related to enzymatics hydrolysis of substrates are different from those reported for fungal enzymes in which case,the end products have been reported as the mono, di, and tri-galacturonates [9, 26, 48, 49] and suggest further studies for better understanding of the mechanism of enzyme hydrolyses at transcriptome and protemoe levels.

## Conclusions

An endo-PGase gene isolated from *Pectobacterium carotovorum* was successfully cloned and expressed in *Bacillus subtilis*. The desired PCR product was 1209bp which encoded an open reading frame of 402 amino acids. Recombinant proteins showed an estimated molecular weight of 48 kDa. Optimal activity of recombinant endo-PGase was found at 40°C and pH 5.0. Potassium chloride increased the enzyme activity while EDTA, Zn^++^ and Ca^++^, strongly inhibited the activity. The chromatographic analysis of enzymatic hydrolysates of polygalacturonic acid (PGA) and pectin substrates using HPLC and TLC revealed tri and tetra-galacturonates as the end products of recombinant endo-PGase hydrolysis. The enzyme can be produced on a very simple medium with IPTG induction only. The mode of action of recombinant enzyme was almost similar to other endo-PGase from yeast and fungi. As compared with yeast and fungal expression systems, *Bacillus subtilis* might be safer for commercial enzyme preparations being non-pathogenic and free of endotoxins. Overall, the current study will help in future research works focused to further optimizing the catalytic performance of endopolyglacturonse for processing of pectin-rich materials used in food and fibre industry.

## Future prospects and suggestions

It is reported that *B*. *subtilis* requires a detailed study of promoters which regulate the initial mRNA transcript levels in order to get the enhanced and properly adjusted expression levels of a gene being metabolically engineered into it [50]. Hence, for the heterologous expression of genes in *B*. *subtilis*, consecutive and inductive promoters are generally utilized [51]. Additionally, expression levels of a protein are also influenced by the ribosomal binding site strength and can further be manipulated by using plasmids with different copy numbers [51, 52]. Expression feature varies from gene to gene and therefore, in metabolic engineering, the use of promoters with variable strengths is crucial for appropriate adjustment of their expression level specific to a metabolic pathway [53]. *B*. *subtilis* offers a broad array of well-developed genetic tools, plasmid expression systems, and promoters which can be used in protein expression, metabolic engineering and synthetic biology. *B*. *subtilis* has a single cell membrane, which differ it from *E*. *coli* and thus, can facilitate protein secretion, simplify downstream processing, and reduce the production costs for the production of industrial enzymes and medicinal proteins. Though, *B*. *subtilis* manifests several valuable applications, it has received much less attraction than *E*. *coli* in metabolic engineering. One major hindrance is the lower efficiency of plasmids construction in *B*. *subtilis* compared with *E*. *coli*. To solve this problem, engineered cells of *B*. *subtilis* can be developed for the direct construction of plasmids in future. Moreover, the high recombination frequency of *B*. *subtilis* offers various advantages for genome editing tools development which will be the topic of future interest for our research group.

It has also been reported that pectinolytic enzymes can induce the biotic stress resistance in plants. Additionally, inactive pectinase could be applied as a bio -pesticide for green food production and will lead to the environment protection and food safety [42] in changing climates round the globe.

If geneticist, molecular biologists and metabolic engineers collaboratively study *B*. *subtilis*, more and more methods, tools and technologies will emerge and will be applied in the future. Consequently, potential use of *B*. *subtilis* in industrial applications will be further enhanced [52].
