# Expression of Concern: Metabolic engineering of *Bacillus subtilis* with an endopolygalacturonase gene isolated from *Pectobacterium. carotovorum*; a plant pathogenic bacterial strain

**DOI:** 10.1371/journal.pone.0351798

**Published:** 2026-06-17

**Authors:** The PLOS One Editors

The *PLOS One* Editors issue this Expression of Concern for this article [1] because of concerns identified about the peer review process. Readers are advised to interpret the article [1] with caution.
